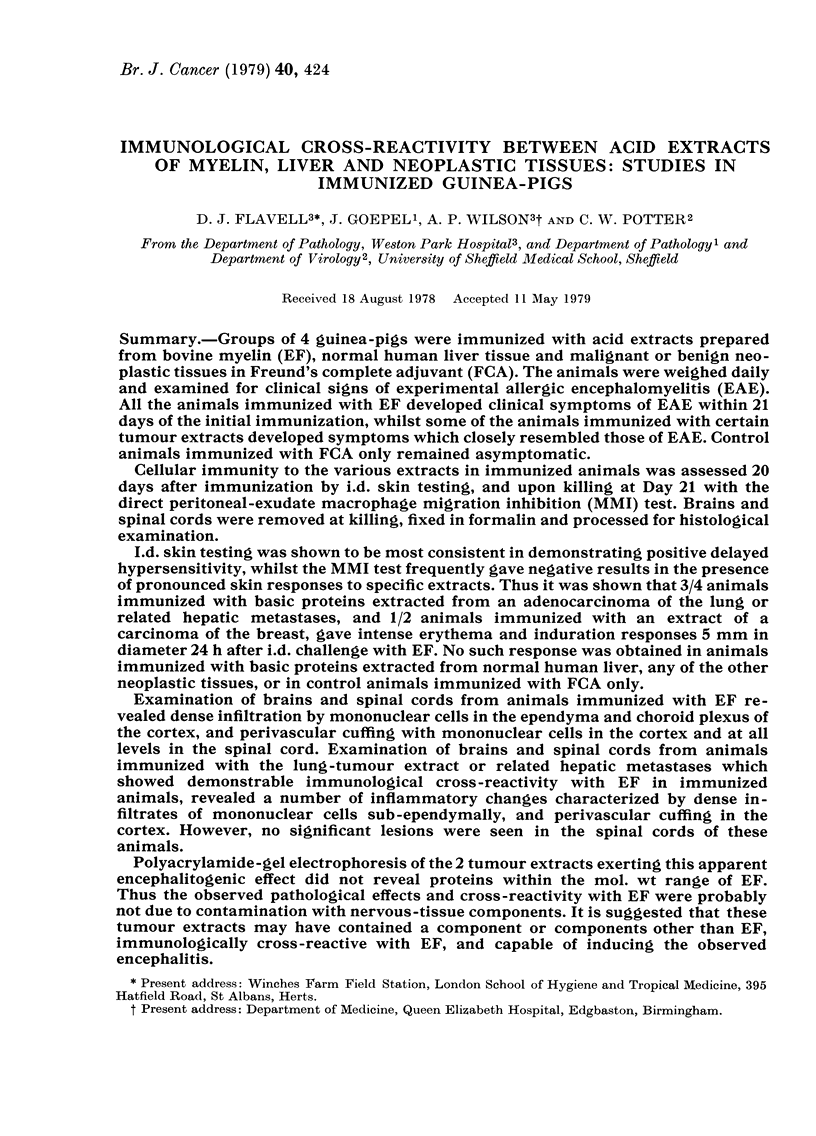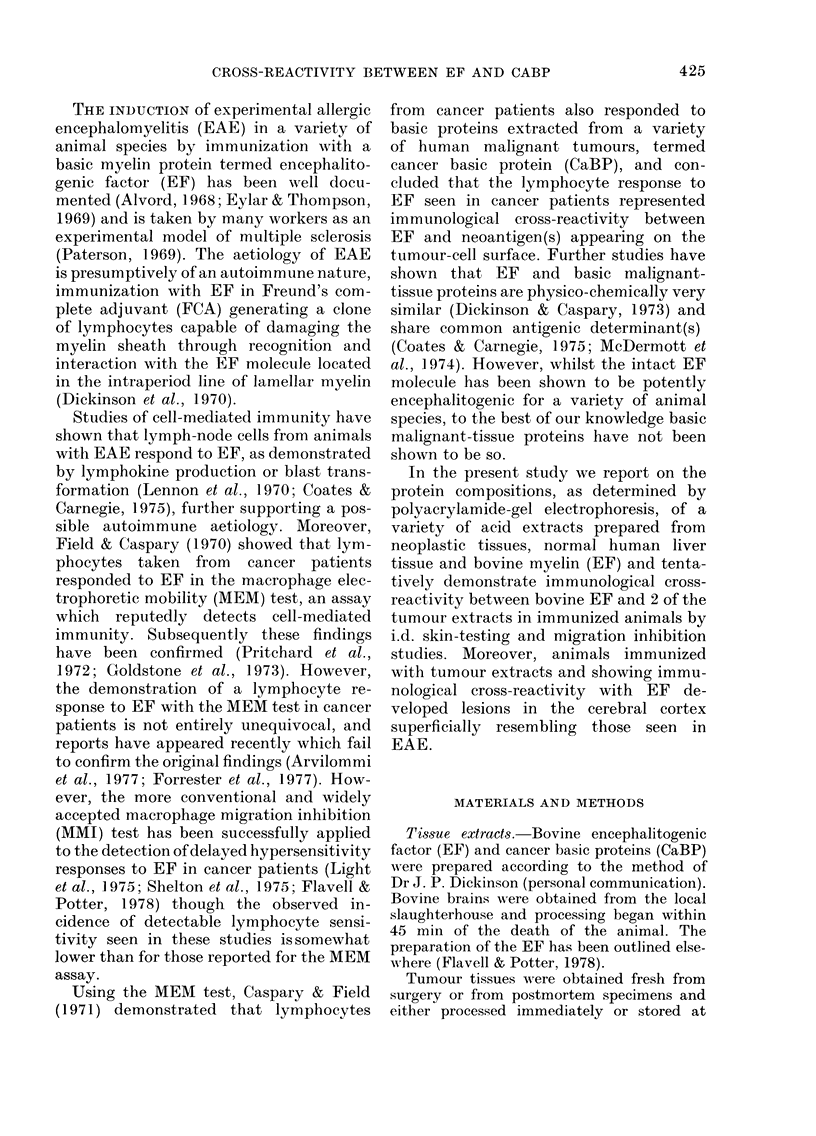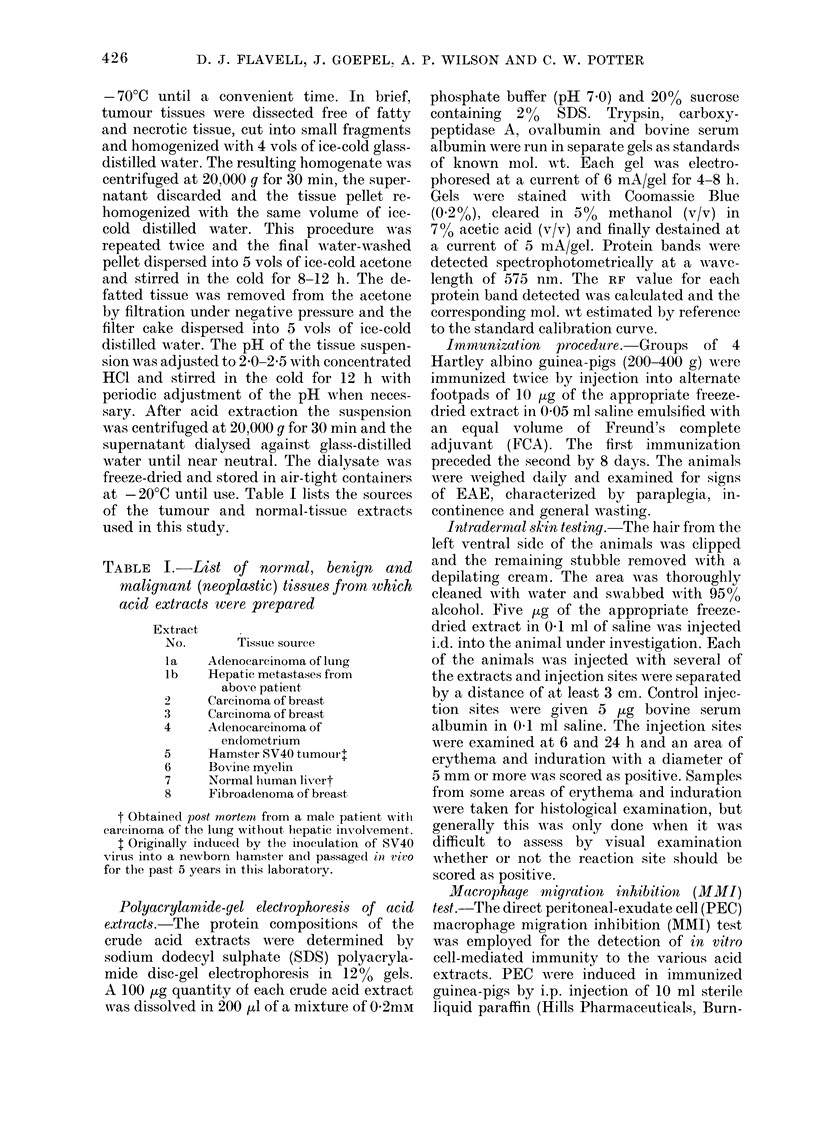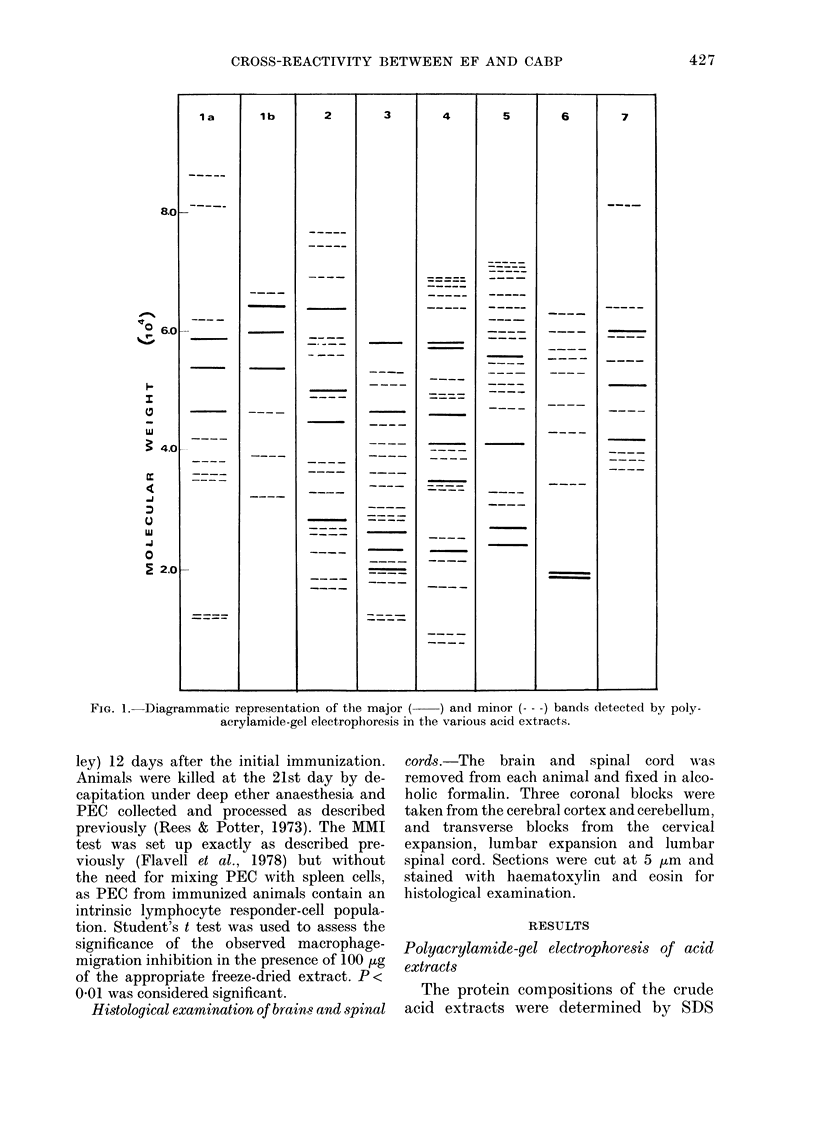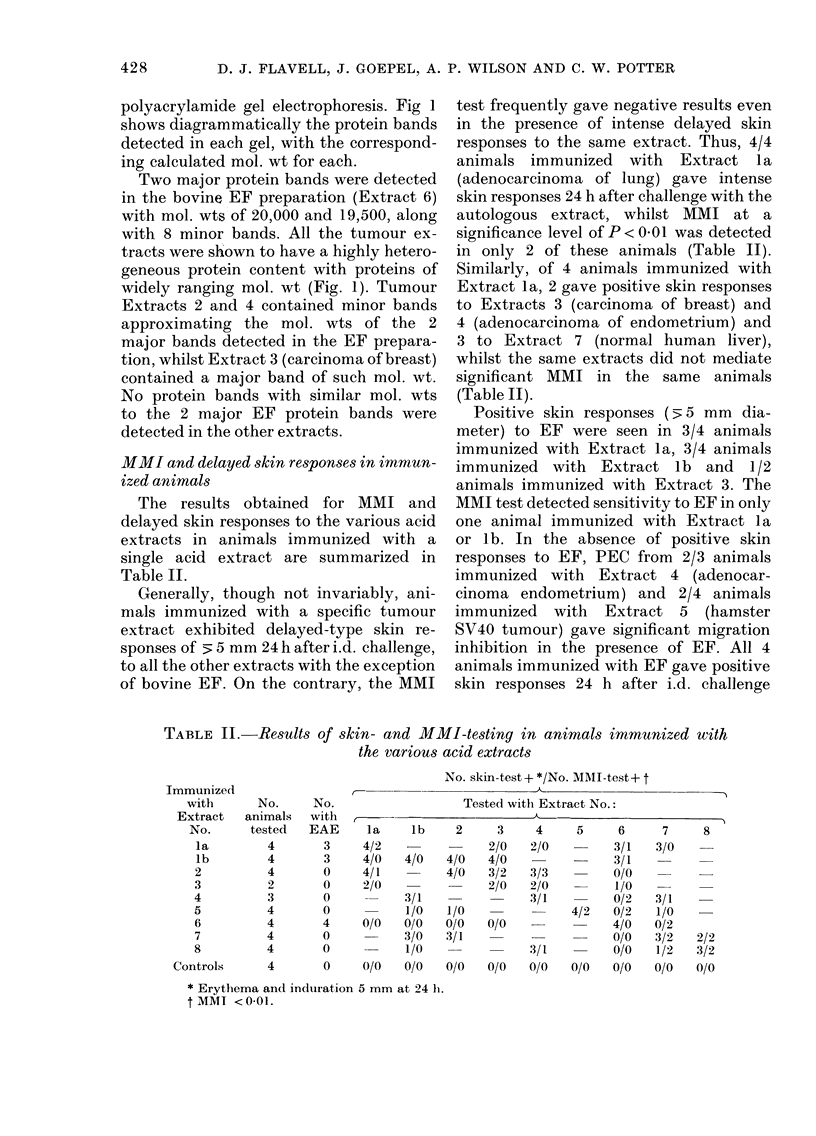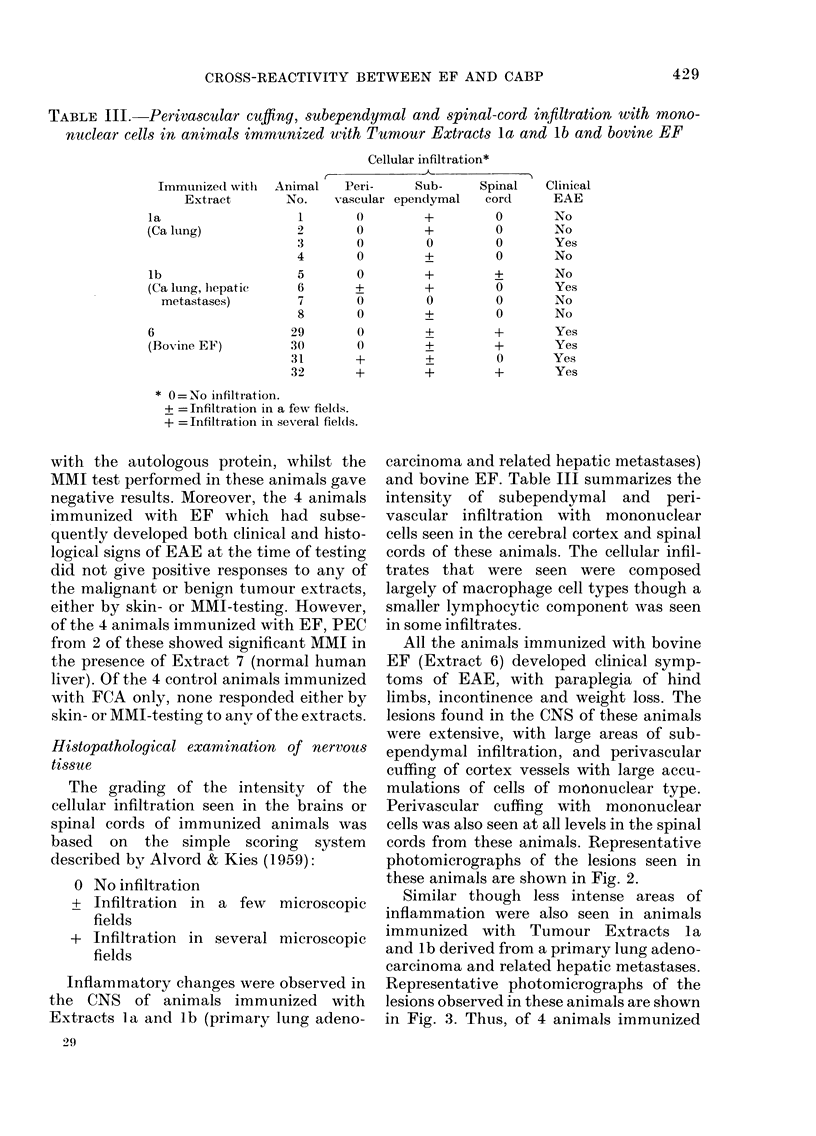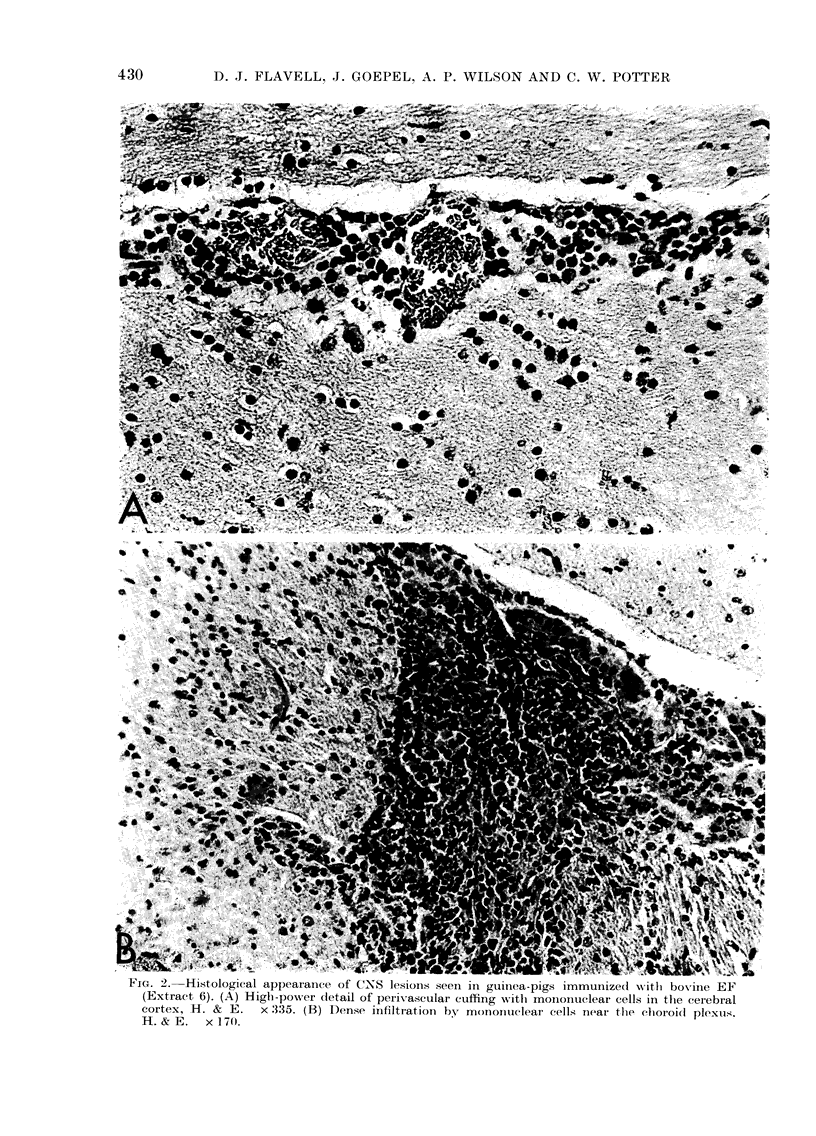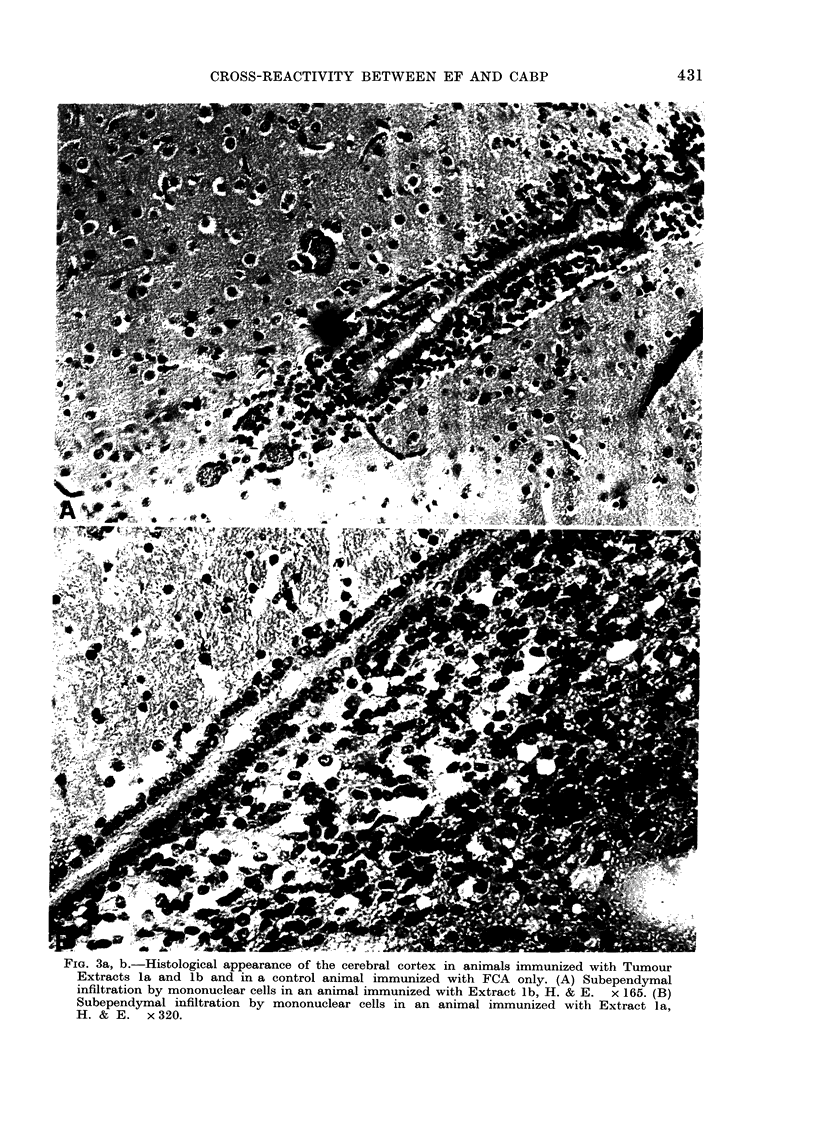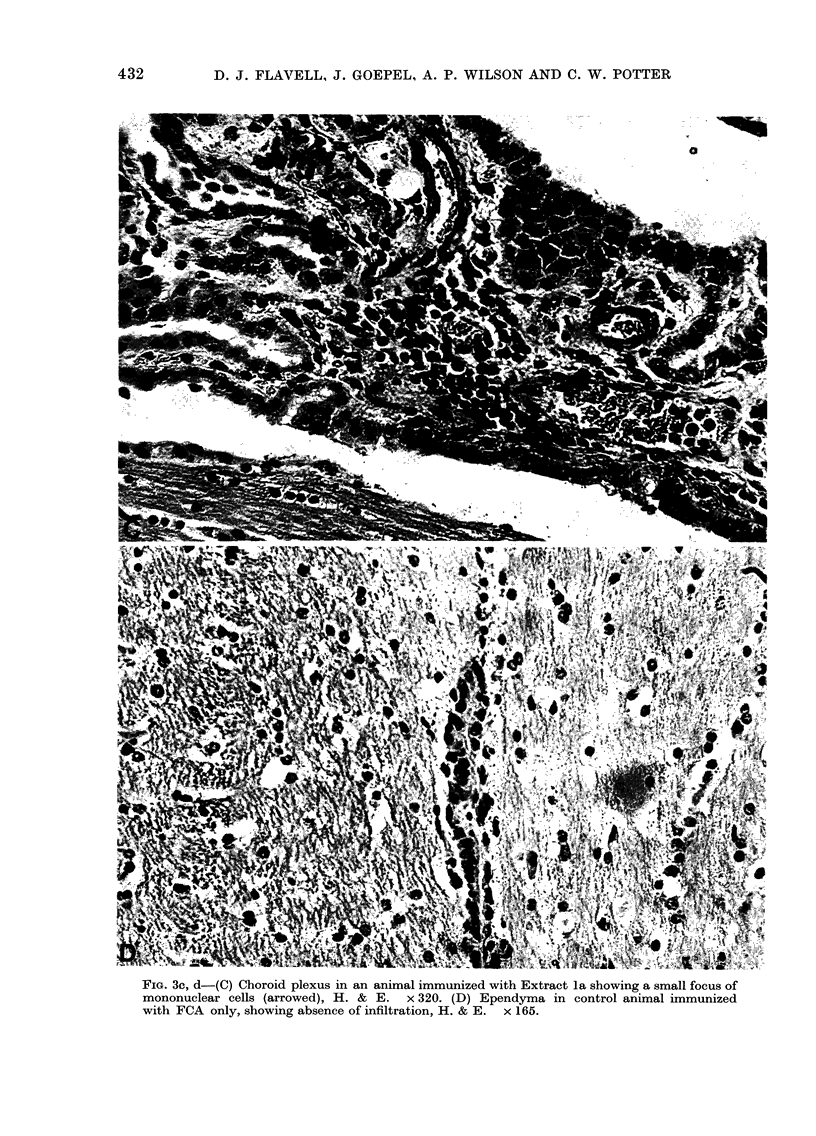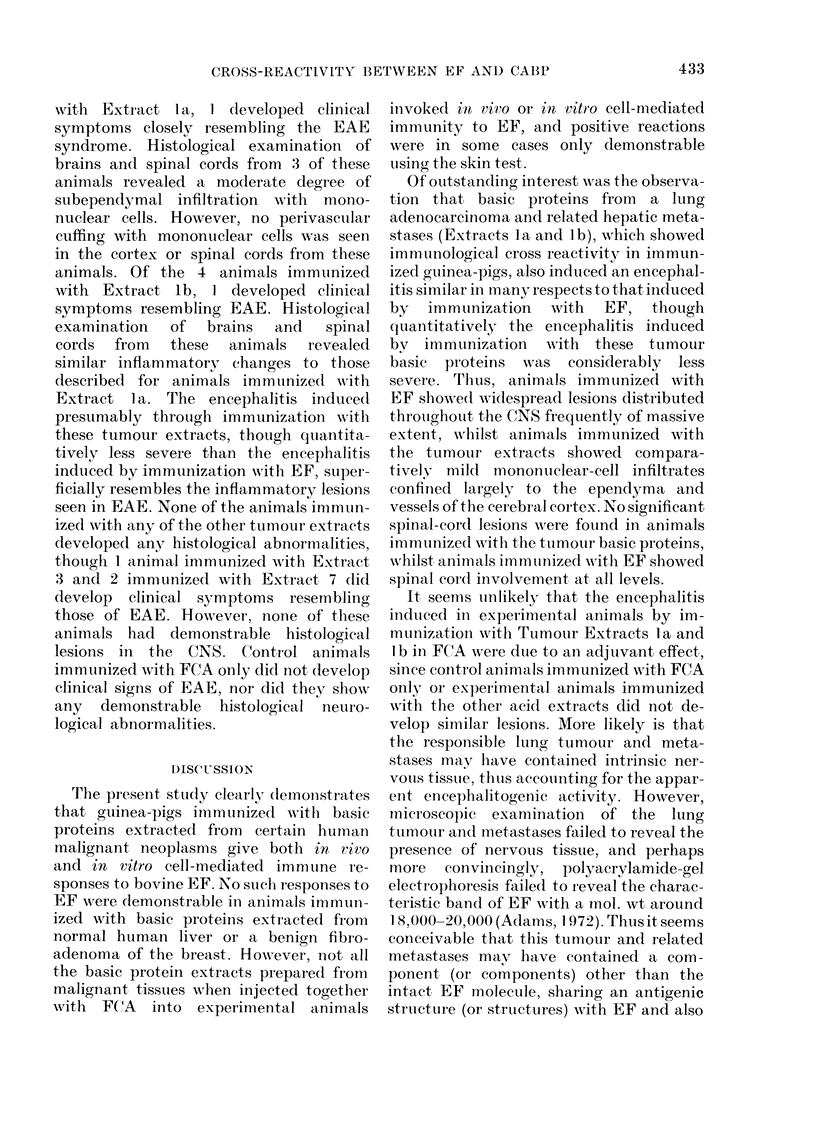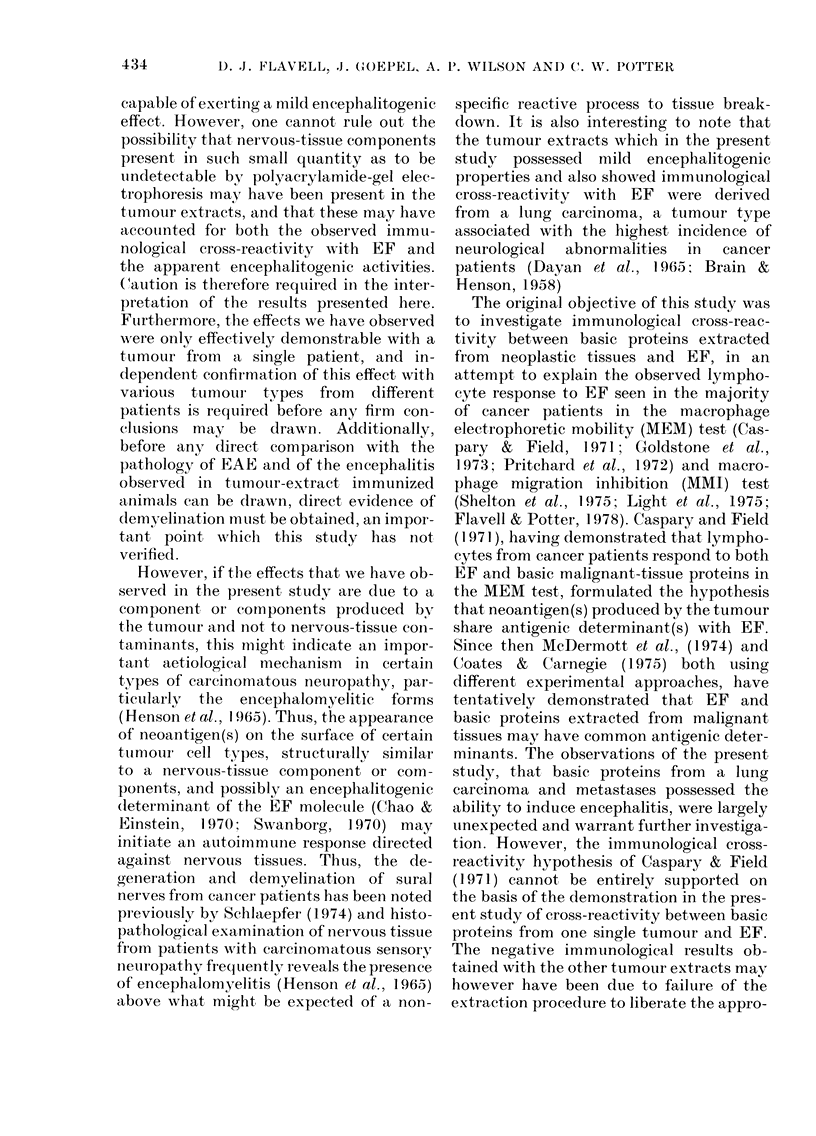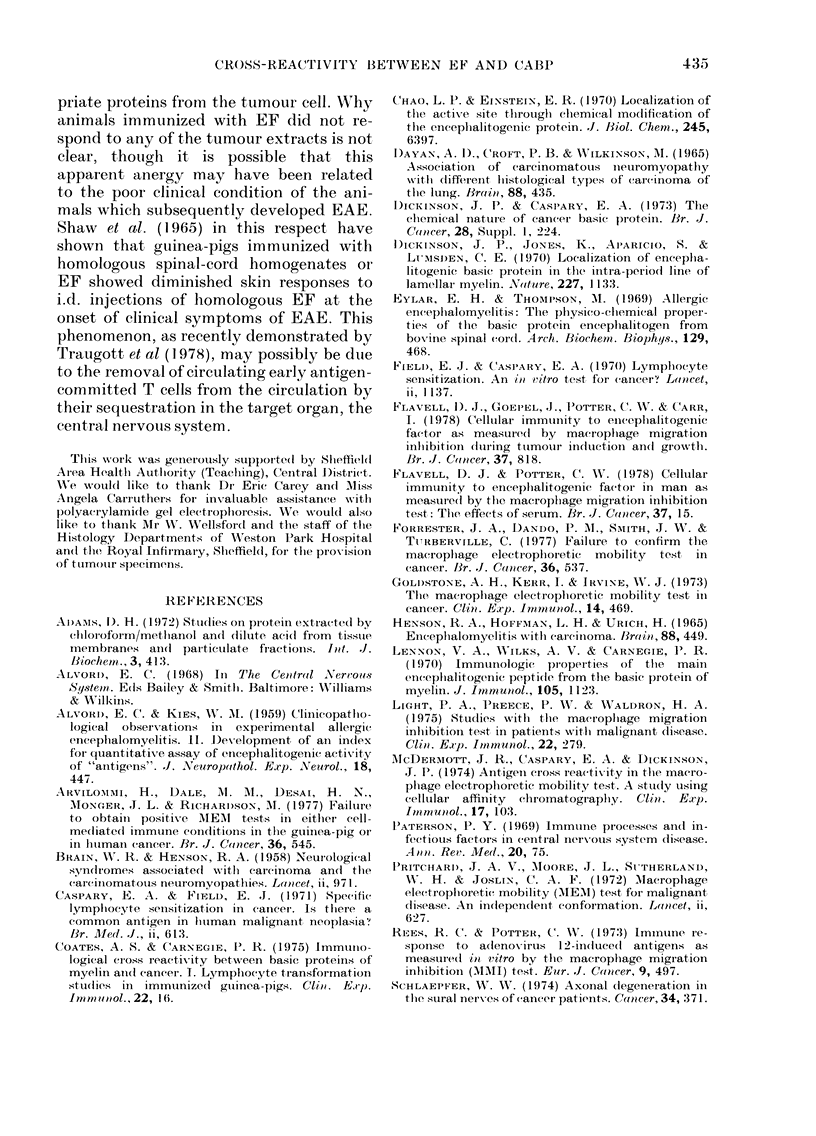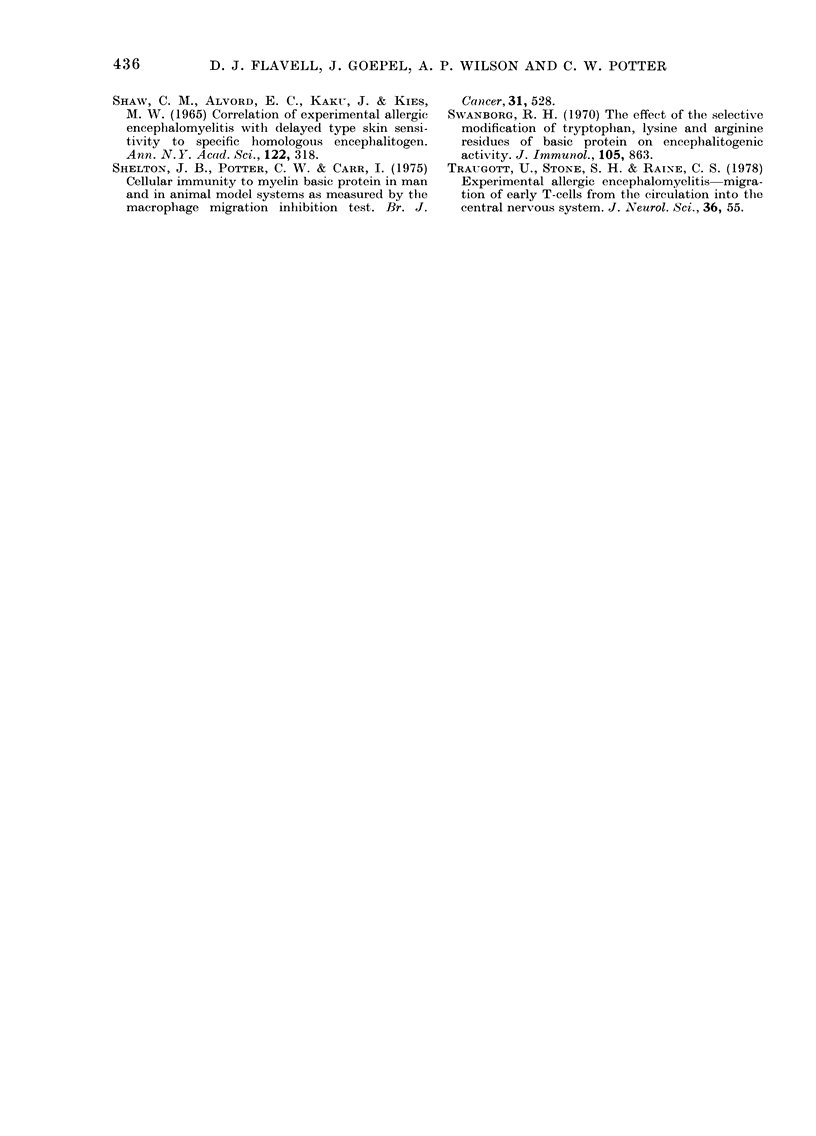# Immunological cross-reactivity between acid extracts of myelin, liver and neoplastic tissues: studies in immunized guinea-pigs.

**DOI:** 10.1038/bjc.1979.198

**Published:** 1979-09

**Authors:** D. J. Flavell, J. Goepel, A. P. Wilson, C. W. Potter

## Abstract

**Images:**


*Br. J. Cancer* (1979) **40**, 424

### IMMUNOLOGICAL CROSS-REACTIVITY BETWEEN ACID EXTRACTS OF MYELIN, LIVER AND NEOPLASTIC TISSUES: STUDIES IN IMMUNIZED GUINEA-PIGS

D. J. FLAVELL[3]*, J. GOEPEL[1], A. P. WILSON[3]† AND C. W. POTTER[2]

*From the Department of Pathology, Weston Park Hospital*[3], *and Department of Pathology*[1] *and Department of Virology*[2], *University of Sheffield Medical School, Sheffield*

Received 18 August 1978  Accepted 11 May 1979

**Summary.—Groups of 4 guinea-pigs were immunized with acid extracts prepared from bovine myelin (EF), normal human liver tissue and malignant or benign neoplastic tissues in Freund's complete adjuvant (FCA). The animals were weighed daily and examined for clinical signs of experimental allergic encephalomyelitis (EAE). All the animals immunized with EF developed clinical symptoms of EAE within 21 days of the initial immunization, whilst some of the animals immunized with certain tumour extracts developed symptoms which closely resembled those of EAE. Control animals immunized with FCA only remained asymptomatic.**

**Cellular immunity to the various extracts in immunized animals was assessed 20 days after immunization by i.d. skin testing, and upon killing at Day 21 with the direct peritoneal-exudate macrophage migration inhibition (MMI) test. Brains and spinal cords were removed at killing, fixed in formalin and processed for histological examination.**

**I.d. skin testing was shown to be most consistent in demonstrating positive delayed hypersensitivity, whilst the MMI test frequently gave negative results in the presence of pronounced skin responses to specific extracts. Thus it was shown that 3/4 animals immunized with basic proteins extracted from an adenocarcinoma of the lung or related hepatic metastases, and 1/2 animals immunized with an extract of a carcinoma of the breast, gave intense erythema and induration responses 5 mm in diameter 24 h after i.d. challenge with EF. No such response was obtained in animals immunized with basic proteins extracted from normal human liver, any of the other neoplastic tissues, or in control animals immunized with FCA only.**

**Examination of brains and spinal cords from animals immunized with EF revealed dense infiltration by mononuclear cells in the ependyma and choroid plexus of the cortex, and perivascular cuffing with mononuclear cells in the cortex and at all levels in the spinal cord. Examination of brains and spinal cords from animals immunized with the lung-tumour extract or related hepatic metastases which showed demonstrable immunological cross-reactivity with EF in immunized animals, revealed a number of inflammatory changes characterized by dense infiltrates of mononuclear cells sub-ependymally, and perivascular cuffing in the cortex. However, no significant lesions were seen in the spinal cords of these animals.**

**Polyacrylamide-gel electrophoresis of the 2 tumour extracts exerting this apparent encephalitogenic effect did not reveal proteins within the mol. wt range of EF. Thus the observed pathological effects and cross-reactivity with EF were probably not due to contamination with nervous-tissue components. It is suggested that these tumour extracts may have contained a component or components other than EF, immunologically cross-reactive with EF, and capable of inducing the observed encephalitis.**

* Present address: Winches Farm Field Station, London School of Hygiene and Tropical Medicine, 395 Hatfield Road, St Albans, Herts.

† Present address: Department of Medicine, Queen Elizabeth Hospital, Edgbaston, Birmingham.

THE INDUCTION of experimental allergic encephalomyelitis (EAE) in a variety of animal species by immunization with a basic myelin protein termed encephalitogenic factor (EF) has been well documented (Alvord, 1968; Eylar & Thompson, 1969) and is taken by many workers as an experimental model of multiple sclerosis (Paterson, 1969). The aetiology of EAE is presumptively of an autoimmune nature, immunization with EF in Freund's complete adjuvant (FCA) generating a clone of lymphocytes capable of damaging the myelin sheath through recognition and interaction with the EF molecule located in the intraperiod line of lamellar myelin (Dickinson *et al.*, 1970).

Studies of cell-mediated immunity have shown that lymph-node cells from animals with EAE respond to EF, as demonstrated by lymphokine production or blast transformation (Lennon *et al.*, 1970; Coates & Carnegie, 1975), further supporting a possible autoimmune aetiology. Moreover, Field & Caspary (1970) showed that lymphocytes taken from cancer patients responded to EF in the macrophage electrophoretic mobility (MEM) test, an assay which reputedly detects cell-mediated immunity. Subsequently these findings have been confirmed (Pritchard *et al.*, 1972; Goldstone *et al.*, 1973). However, the demonstration of a lymphocyte response to EF with the MEM test in cancer patients is not entirely unequivocal, and reports have appeared recently which fail to confirm the original findings (Arvilommi *et al.*, 1977; Forrester *et al.*, 1977). However, the more conventional and widely accepted macrophage migration inhibition (MMI) test has been successfully applied to the detection of delayed hypersensitivity responses to EF in cancer patients (Light *et al.*, 1975; Shelton *et al.*, 1975; Flavell & Potter, 1978) though the observed incidence of detectable lymphocyte sensitivity seen in these studies is somewhat lower than for those reported for the MEM assay.

Using the MEM test, Caspary & Field (1971) demonstrated that lymphocytes from cancer patients also responded to basic proteins extracted from a variety of human malignant tumours, termed cancer basic protein (CaBP), and concluded that the lymphocyte response to EF seen in cancer patients represented immunological cross-reactivity between EF and neoantigen(s) appearing on the tumour-cell surface. Further studies have shown that EF and basic malignant-tissue proteins are physico-chemically very similar (Dickinson & Caspary, 1973) and share common antigenic determinant(s) (Coates & Carnegie, 1975; McDermott *et al.*, 1974). However, whilst the intact EF molecule has been shown to be potently encephalitogenic for a variety of animal species, to the best of our knowledge basic malignant-tissue proteins have not been shown to be so.

In the present study we report on the protein compositions, as determined by polyacrylamide-gel electrophoresis, of a variety of acid extracts prepared from neoplastic tissues, normal human liver tissue and bovine myelin (EF) and tentatively demonstrate immunological cross-reactivity between bovine EF and 2 of the tumour extracts in immunized animals by i.d. skin-testing and migration inhibition studies. Moreover, animals immunized with tumour extracts and showing immunological cross-reactivity with EF developed lesions in the cerebral cortex superficially resembling those seen in EAE.

### MATERIALS AND METHODS

*Tissue extracts.*—Bovine encephalitogenic factor (EF) and cancer basic proteins (CaBP) were prepared according to the method of Dr J. P. Dickinson (personal communication). Bovine brains were obtained from the local slaughterhouse and processing began within 45 min of the death of the animal. The preparation of the EF has been outlined elsewhere (Flavell & Potter, 1978).

Tumour tissues were obtained fresh from surgery or from postmortem specimens and either processed immediately or stored at

$-70°C$ until a convenient time. In brief, tumour tissues were dissected free of fatty and necrotic tissue, cut into small fragments and homogenized with 4 vols of ice-cold glass-distilled water. The resulting homogenate was centrifuged at 20,000 *g* for 30 min, the supernatant discarded and the tissue pellet re-homogenized with the same volume of ice-cold distilled water. This procedure was repeated twice and the final water-washed pellet dispersed into 5 vols of ice-cold acetone and stirred in the cold for 8–12 h. The defatted tissue was removed from the acetone by filtration under negative pressure and the filter cake dispersed into 5 vols of ice-cold distilled water. The pH of the tissue suspension was adjusted to 2·0–2·5 with concentrated HCl and stirred in the cold for 12 h with periodic adjustment of the pH when necessary. After acid extraction the suspension was centrifuged at 20,000 *g* for 30 min and the supernatant dialysed against glass-distilled water until near neutral. The dialysate was freeze-dried and stored in air-tight containers at $-20°C$ until use. Table I lists the sources of the tumour and normal-tissue extracts used in this study.

TABLE I.—*List of normal, benign and malignant (neoplastic) tissues from which acid extracts were prepared*

| Extract No. | Tissue source |
|---|---|
| 1a | Adenocarcinoma of lung |
| 1b | Hepatic metastases from above patient |
| 2 | Carcinoma of breast |
| 3 | Carcinoma of breast |
| 4 | Adenocarcinoma of endometrium |
| 5 | Hamster SV40 tumour‡ |
| 6 | Bovine myelin |
| 7 | Normal human liver† |
| 8 | Fibroadenoma of breast |

† Obtained *post mortem* from a male patient with carcinoma of the lung without hepatic involvement.

‡ Originally induced by the inoculation of SV40 virus into a newborn hamster and passaged *in vivo* for the past 5 years in this laboratory.

*Polyacrylamide-gel electrophoresis of acid extracts.*—The protein compositions of the crude acid extracts were determined by sodium dodecyl sulphate (SDS) polyacrylamide disc-gel electrophoresis in 12% gels. A 100 $\mu$g quantity of each crude acid extract was dissolved in 200 $\mu$l of a mixture of 0·2mM phosphate buffer (pH 7·0) and 20% sucrose containing 2% SDS. Trypsin, carboxypeptidase A, ovalbumin and bovine serum albumin were run in separate gels as standards of known mol. wt. Each gel was electrophoresed at a current of 6 mA/gel for 4–8 h. Gels were stained with Coomassie Blue (0·2%), cleared in 5% methanol (v/v) in 7% acetic acid (v/v) and finally destained at a current of 5 mA/gel. Protein bands were detected spectrophotometrically at a wavelength of 575 nm. The RF value for each protein band detected was calculated and the corresponding mol. wt estimated by reference to the standard calibration curve.

*Immunization procedure.*—Groups of 4 Hartley albino guinea-pigs (200–400 g) were immunized twice by injection into alternate footpads of 10 $\mu$g of the appropriate freeze-dried extract in 0·05 ml saline emulsified with an equal volume of Freund's complete adjuvant (FCA). The first immunization preceded the second by 8 days. The animals were weighed daily and examined for signs of EAE, characterized by paraplegia, incontinence and general wasting.

*Intradermal skin testing.*—The hair from the left ventral side of the animals was clipped and the remaining stubble removed with a depilating cream. The area was thoroughly cleaned with water and swabbed with 95% alcohol. Five $\mu$g of the appropriate freeze-dried extract in 0·1 ml of saline was injected i.d. into the animal under investigation. Each of the animals was injected with several of the extracts and injection sites were separated by a distance of at least 3 cm. Control injection sites were given 5 $\mu$g bovine serum albumin in 0·1 ml saline. The injection sites were examined at 6 and 24 h and an area of erythema and induration with a diameter of 5 mm or more was scored as positive. Samples from some areas of erythema and induration were taken for histological examination, but generally this was only done when it was difficult to assess by visual examination whether or not the reaction site should be scored as positive.

*Macrophage migration inhibition (MMI) test.*—The direct peritoneal-exudate cell (PEC) macrophage migration inhibition (MMI) test was employed for the detection of *in vitro* cell-mediated immunity to the various acid extracts. PEC were induced in immunized guinea-pigs by i.p. injection of 10 ml sterile liquid paraffin (Hills Pharmaceuticals, Burn-

FIG. 1.—Diagrammatic representation of the major (———) and minor (- - -) bands detected by polyacrylamide-gel electrophoresis in the various acid extracts.

ley) 12 days after the initial immunization. Animals were killed at the 21st day by decapitation under deep ether anaesthesia and PEC collected and processed as described previously (Rees & Potter, 1973). The MMI test was set up exactly as described previously (Flavell *et al.*, 1978) but without the need for mixing PEC with spleen cells, as PEC from immunized animals contain an intrinsic lymphocyte responder-cell population. Student's *t* test was used to assess the significance of the observed macrophage-migration inhibition in the presence of 100 $\mu$g of the appropriate freeze-dried extract. $P <$ 0·01 was considered significant.

*Histological examination of brains and spinal cords.*—The brain and spinal cord was removed from each animal and fixed in alcoholic formalin. Three coronal blocks were taken from the cerebral cortex and cerebellum, and transverse blocks from the cervical expansion, lumbar expansion and lumbar spinal cord. Sections were cut at 5 $\mu$m and stained with haematoxylin and eosin for histological examination.

### RESULTS

### *Polyacrylamide-gel electrophoresis of acid extracts*

The protein compositions of the crude acid extracts were determined by SDS

polyacrylamide gel electrophoresis. Fig 1 shows diagrammatically the protein bands detected in each gel, with the corresponding calculated mol. wt for each.

Two major protein bands were detected in the bovine EF preparation (Extract 6) with mol. wts of 20,000 and 19,500, along with 8 minor bands. All the tumour extracts were shown to have a highly heterogeneous protein content with proteins of widely ranging mol. wt (Fig. 1). Tumour Extracts 2 and 4 contained minor bands approximating the mol. wts of the 2 major bands detected in the EF preparation, whilst Extract 3 (carcinoma of breast) contained a major band of such mol. wt. No protein bands with similar mol. wts to the 2 major EF protein bands were detected in the other extracts.

*MMI and delayed skin responses in immunized animals*

The results obtained for MMI and delayed skin responses to the various acid extracts in animals immunized with a single acid extract are summarized in Table II.

Generally, though not invariably, animals immunized with a specific tumour extract exhibited delayed-type skin responses of $\geq 5$ mm 24 h after i.d. challenge, to all the other extracts with the exception of bovine EF. On the contrary, the MMI test frequently gave negative results even in the presence of intense delayed skin responses to the same extract. Thus, 4/4 animals immunized with Extract 1a (adenocarcinoma of lung) gave intense skin responses 24 h after challenge with the autologous extract, whilst MMI at a significance level of $P < 0 \cdot 01$ was detected in only 2 of these animals (Table II). Similarly, of 4 animals immunized with Extract 1a, 2 gave positive skin responses to Extracts 3 (carcinoma of breast) and 4 (adenocarcinoma of endometrium) and 3 to Extract 7 (normal human liver), whilst the same extracts did not mediate significant MMI in the same animals (Table II).

Positive skin responses ($\geq 5$ mm diameter) to EF were seen in 3/4 animals immunized with Extract 1a, 3/4 animals immunized with Extract 1b and 1/2 animals immunized with Extract 3. The MMI test detected sensitivity to EF in only one animal immunized with Extract 1a or 1b. In the absence of positive skin responses to EF, PEC from 2/3 animals immunized with Extract 4 (adenocarcinoma endometrium) and 2/4 animals immunized with Extract 5 (hamster SV40 tumour) gave significant migration inhibition in the presence of EF. All 4 animals immunized with EF gave positive skin responses 24 h after i.d. challenge

TABLE II.—*Results of skin- and MMI-testing in animals immunized with the various acid extracts*

| Immunized with Extract No. | No. animals tested | No. with EAE | No. skin-test + */No. MMI-test + † Tested with Extract No.: 1a | 1b | 2 | 3 | 4 | 5 | 6 | 7 | 8 |
|---|---|---|---|---|---|---|---|---|---|---|---|
| 1a | 4 | 3 | 4/2 | — | — | 2/0 | 2/0 | — | 3/1 | 3/0 | — |
| 1b | 4 | 3 | 4/0 | 4/0 | 4/0 | 4/0 | — | — | 3/1 | — | — |
| 2 | 4 | 0 | 4/1 | — | 4/0 | 3/2 | 3/3 | — | 0/0 | — | — |
| 3 | 2 | 0 | 2/0 | — | — | 2/0 | 2/0 | — | 1/0 | — | — |
| 4 | 3 | 0 | — | 3/1 | — | — | 3/1 | — | 0/2 | 3/1 | — |
| 5 | 4 | 0 | — | 1/0 | 1/0 | — | — | 4/2 | 0/2 | 1/0 | — |
| 6 | 4 | 4 | 0/0 | 0/0 | 0/0 | 0/0 | — | — | 4/0 | 0/2 | |
| 7 | 4 | 0 | — | 3/0 | 3/1 | — | — | — | 0/0 | 3/2 | 2/2 |
| 8 | 4 | 0 | — | 1/0 | — | — | 3/1 | — | 0/0 | 1/2 | 3/2 |
| Controls | 4 | 0 | 0/0 | 0/0 | 0/0 | 0/0 | 0/0 | 0/0 | 0/0 | 0/0 | 0/0 |

* Erythema and induration 5 mm at 24 h.

† MMI < 0·01.

TABLE III.—*Perivascular cuffing, subependymal and spinal-cord infiltration with mononuclear cells in animals immunized with Tumour Extracts* 1*a and* 1*b and bovine EF*

| Immunized with Extract | Animal No. | Cellular infiltration* Perivascular | Subependymal | Spinal cord | Clinical EAE |
|---|---|---|---|---|---|
| 1a | 1 | 0 | + | 0 | No |
| (Ca lung) | 2 | 0 | + | 0 | No |
| | 3 | 0 | 0 | 0 | Yes |
| | 4 | 0 | ± | 0 | No |
| 1b | 5 | 0 | + | ± | No |
| (Ca lung, hepatic metastases) | 6 | ± | + | 0 | Yes |
| | 7 | 0 | 0 | 0 | No |
| | 8 | 0 | ± | 0 | No |
| 6 | 29 | 0 | ± | + | Yes |
| (Bovine EF) | 30 | 0 | ± | + | Yes |
| | 31 | + | ± | 0 | Yes |
| | 32 | + | + | + | Yes |

* 0 = No infiltration.
± = Infiltration in a few fields.
\+ = Infiltration in several fields.

with the autologous protein, whilst the MMI test performed in these animals gave negative results. Moreover, the 4 animals immunized with EF which had subsequently developed both clinical and histological signs of EAE at the time of testing did not give positive responses to any of the malignant or benign tumour extracts, either by skin- or MMI-testing. However, of the 4 animals immunized with EF, PEC from 2 of these showed significant MMI in the presence of Extract 7 (normal human liver). Of the 4 control animals immunized with FCA only, none responded either by skin- or MMI-testing to any of the extracts.

*Histopathological examination of nervous tissue*

The grading of the intensity of the cellular infiltration seen in the brains or spinal cords of immunized animals was based on the simple scoring system described by Alvord & Kies (1959):

0 No infiltration
± Infiltration in a few microscopic fields
\+ Infiltration in several microscopic fields

Inflammatory changes were observed in the CNS of animals immunized with Extracts 1a and 1b (primary lung adenocarcinoma and related hepatic metastases) and bovine EF. Table III summarizes the intensity of subependymal and perivascular infiltration with mononuclear cells seen in the cerebral cortex and spinal cords of these animals. The cellular infiltrates that were seen were composed largely of macrophage cell types though a smaller lymphocytic component was seen in some infiltrates.

All the animals immunized with bovine EF (Extract 6) developed clinical symptoms of EAE, with paraplegia of hind limbs, incontinence and weight loss. The lesions found in the CNS of these animals were extensive, with large areas of subependymal infiltration, and perivascular cuffing of cortex vessels with large accumulations of cells of mononuclear type. Perivascular cuffing with mononuclear cells was also seen at all levels in the spinal cords from these animals. Representative photomicrographs of the lesions seen in these animals are shown in Fig. 2.

Similar though less intense areas of inflammation were also seen in animals immunized with Tumour Extracts 1a and 1b derived from a primary lung adenocarcinoma and related hepatic metastases. Representative photomicrographs of the lesions observed in these animals are shown in Fig. 3. Thus, of 4 animals immunized

FIG. 2.—Histological appearance of CNS lesions seen in guinea-pigs immunized with bovine EF (Extract 6). (A) High-power detail of perivascular cuffing with mononuclear cells in the cerebral cortex, H. & E. ×335. (B) Dense infiltration by mononuclear cells near the choroid plexus, H. & E. ×170.

FIG. 3a, b.—Histological appearance of the cerebral cortex in animals immunized with Tumour Extracts 1a and 1b and in a control animal immunized with FCA only. (A) Subependymal infiltration by mononuclear cells in an animal immunized with Extract 1b, H. & E. ×165. (B) Subependymal infiltration by mononuclear cells in an animal immunized with Extract 1a, H. & E. ×320.

FIG. 3c, d—(C) Choroid plexus in an animal immunized with Extract 1a showing a small focus of mononuclear cells (arrowed), H. & E. ×320. (D) Ependyma in control animal immunized with FCA only, showing absence of infiltration, H. & E. ×165.

with Extract 1a, 1 developed clinical symptoms closely resembling the EAE syndrome. Histological examination of brains and spinal cords from 3 of these animals revealed a moderate degree of subependymal infiltration with mononuclear cells. However, no perivascular cuffing with mononuclear cells was seen in the cortex or spinal cords from these animals. Of the 4 animals immunized with Extract 1b, 1 developed clinical symptoms resembling EAE. Histological examination of brains and spinal cords from these animals revealed similar inflammatory changes to those described for animals immunized with Extract 1a. The encephalitis induced presumably through immunization with these tumour extracts, though quantitatively less severe than the encephalitis induced by immunization with EF, superficially resembles the inflammatory lesions seen in EAE. None of the animals immunized with any of the other tumour extracts developed any histological abnormalities, though 1 animal immunized with Extract 3 and 2 immunized with Extract 7 did develop clinical symptoms resembling those of EAE. However, none of these animals had demonstrable histological lesions in the CNS. Control animals immunized with FCA only did not develop clinical signs of EAE, nor did they show any demonstrable histological neurological abnormalities.

### DISCUSSION

The present study clearly demonstrates that guinea-pigs immunized with basic proteins extracted from certain human malignant neoplasms give both *in vivo* and *in vitro* cell-mediated immune responses to bovine EF. No such responses to EF were demonstrable in animals immunized with basic proteins extracted from normal human liver or a benign fibroadenoma of the breast. However, not all the basic protein extracts prepared from malignant tissues when injected together with FCA into experimental animals invoked *in vivo* or *in vitro* cell-mediated immunity to EF, and positive reactions were in some cases only demonstrable using the skin test.

Of outstanding interest was the observation that basic proteins from a lung adenocarcinoma and related hepatic metastases (Extracts 1a and 1b), which showed immunological cross reactivity in immunized guinea-pigs, also induced an encephalitis similar in many respects to that induced by immunization with EF, though quantitatively the encephalitis induced by immunization with these tumour basic proteins was considerably less severe. Thus, animals immunized with EF showed widespread lesions distributed throughout the CNS frequently of massive extent, whilst animals immunized with the tumour extracts showed comparatively mild mononuclear-cell infiltrates confined largely to the ependyma and vessels of the cerebral cortex. No significant spinal-cord lesions were found in animals immunized with the tumour basic proteins, whilst animals immunized with EF showed spinal cord involvement at all levels.

It seems unlikely that the encephalitis induced in experimental animals by immunization with Tumour Extracts 1a and 1b in FCA were due to an adjuvant effect, since control animals immunized with FCA only or experimental animals immunized with the other acid extracts did not develop similar lesions. More likely is that the responsible lung tumour and metastases may have contained intrinsic nervous tissue, thus accounting for the apparent encephalitogenic activity. However, microscopic examination of the lung tumour and metastases failed to reveal the presence of nervous tissue, and perhaps more convincingly, polyacrylamide-gel electrophoresis failed to reveal the characteristic band of EF with a mol. wt around 18,000–20,000 (Adams, 1972). Thus it seems conceivable that this tumour and related metastases may have contained a component (or components) other than the intact EF molecule, sharing an antigenic structure (or structures) with EF and also capable of exerting a mild encephalitogenic effect. However, one cannot rule out the possibility that nervous-tissue components present in such small quantity as to be undetectable by polyacrylamide-gel electrophoresis may have been present in the tumour extracts, and that these may have accounted for both the observed immunological cross-reactivity with EF and the apparent encephalitogenic activities. Caution is therefore required in the interpretation of the results presented here. Furthermore, the effects we have observed were only effectively demonstrable with a tumour from a single patient, and independent confirmation of this effect with various tumour types from different patients is required before any firm conclusions may be drawn. Additionally, before any direct comparison with the pathology of EAE and of the encephalitis observed in tumour-extract immunized animals can be drawn, direct evidence of demyelination must be obtained, an important point which this study has not verified.

However, if the effects that we have observed in the present study are due to a component or components produced by the tumour and not to nervous-tissue contaminants, this might indicate an important aetiological mechanism in certain types of carcinomatous neuropathy, particularly the encephalomyelitic forms (Henson *et al.*, 1965). Thus, the appearance of neoantigen(s) on the surface of certain tumour cell types, structurally similar to a nervous-tissue component or components, and possibly an encephalitogenic determinant of the EF molecule (Chao & Einstein, 1970; Swanborg, 1970) may initiate an autoimmune response directed against nervous tissues. Thus, the degeneration and demyelination of sural nerves from cancer patients has been noted previously by Schlaepfer (1974) and histopathological examination of nervous tissue from patients with carcinomatous sensory neuropathy frequently reveals the presence of encephalomyelitis (Henson *et al.*, 1965) above what might be expected of a non-specific reactive process to tissue breakdown. It is also interesting to note that the tumour extracts which in the present study possessed mild encephalitogenic properties and also showed immunological cross-reactivity with EF were derived from a lung carcinoma, a tumour type associated with the highest incidence of neurological abnormalities in cancer patients (Dayan *et al.*, 1965; Brain & Henson, 1958)

The original objective of this study was to investigate immunological cross-reactivity between basic proteins extracted from neoplastic tissues and EF, in an attempt to explain the observed lymphocyte response to EF seen in the majority of cancer patients in the macrophage electrophoretic mobility (MEM) test (Caspary & Field, 1971; Goldstone *et al.*, 1973; Pritchard *et al.*, 1972) and macrophage migration inhibition (MMI) test (Shelton *et al.*, 1975; Light *et al.*, 1975; Flavell & Potter, 1978). Caspary and Field (1971), having demonstrated that lymphocytes from cancer patients respond to both EF and basic malignant-tissue proteins in the MEM test, formulated the hypothesis that neoantigen(s) produced by the tumour share antigenic determinant(s) with EF. Since then McDermott *et al.*, (1974) and Coates & Carnegie (1975) both using different experimental approaches, have tentatively demonstrated that EF and basic proteins extracted from malignant tissues may have common antigenic determinants. The observations of the present study, that basic proteins from a lung carcinoma and metastases possessed the ability to induce encephalitis, were largely unexpected and warrant further investigation. However, the immunological cross-reactivity hypothesis of Caspary & Field (1971) cannot be entirely supported on the basis of the demonstration in the present study of cross-reactivity between basic proteins from one single tumour and EF. The negative immunological results obtained with the other tumour extracts may however have been due to failure of the extraction procedure to liberate the appropriate proteins from the tumour cell. Why animals immunized with EF did not respond to any of the tumour extracts is not clear, though it is possible that this apparent anergy may have been related to the poor clinical condition of the animals which subsequently developed EAE. Shaw *et al.* (1965) in this respect have shown that guinea-pigs immunized with homologous spinal-cord homogenates or EF showed diminished skin responses to i.d. injections of homologous EF at the onset of clinical symptoms of EAE. This phenomenon, as recently demonstrated by Traugott *et al* (1978), may possibly be due to the removal of circulating early antigen-committed T cells from the circulation by their sequestration in the target organ, the central nervous system.

This work was generously supported by Sheffield Area Health Authority (Teaching), Central District. We would like to thank Dr Eric Carey and Miss Angela Carruthers for invaluable assistance with polyacrylamide gel electrophoresis. We would also like to thank Mr W. Wellsford and the staff of the Histology Departments of Weston Park Hospital and the Royal Infirmary, Sheffield, for the provision of tumour specimens.

### REFERENCES

ADAMS, D. H. (1972) Studies on protein extracted by chloroform/methanol and dilute acid from tissue membranes and particulate fractions. *Int. J. Biochem.*, **3**, 413.

ALVORD, E. C. (1968) In *The Central Nervous System*. Eds Bailey & Smith. Baltimore: Williams & Wilkins.

ALVORD, E. C. & KIES, W. M. (1959) Clinicopathological observations in experimental allergic encephalomyelitis. II. Development of an index for quantitative assay of encephalitogenic activity of "antigens". *J. Neuropathol. Exp. Neurol.*, **18**, 447.

ARVILOMMI, H., DALE, M. M., DESAI, H. N., MONGER, J. L. & RICHARDSON, M. (1977) Failure to obtain positive MEM tests in either cell-mediated immune conditions in the guinea-pig or in human cancer. *Br. J. Cancer*, **36**, 545.

BRAIN, W. R. & HENSON, R. A. (1958) Neurological syndromes associated with carcinoma and the carcinomatous neuromyopathies. *Lancet*, ii, 971.

CASPARY, E. A. & FIELD, E. J. (1971) Specific lymphocyte sensitization in cancer. Is there a common antigen in human malignant neoplasia? *Br. Med. J.*, ii, 613.

COATES, A. S. & CARNEGIE, P. R. (1975) Immunological cross reactivity between basic proteins of myelin and cancer. I. Lymphocyte transformation studies in immunized guinea-pigs. *Clin. Exp. Immunol.*, **22**, 16.

CHAO, L. P. & EINSTEIN, E. R. (1970) Localization of the active site through chemical modification of the encephalitogenic protein. *J. Biol. Chem.*, **245**, 6397.

DAYAN, A. D., CROFT, P. B. & WILKINSON, M. (1965) Association of carcinomatous neuromyopathy with different histological types of carcinoma of the lung. *Brain*, **88**, 435.

DICKINSON, J. P. & CASPARY, E. A. (1973) The chemical nature of cancer basic protein. *Br. J. Cancer*, **28**, Suppl. 1, 224.

DICKINSON, J. P., JONES, K., APARICIO, S. & LUMSDEN, C. E. (1970) Localization of encephalitogenic basic protein in the intra-period line of lamellar myelin. *Nature*, **227**, 1133.

EYLAR, E. H. & THOMPSON, M. (1969) Allergic encephalomyelitis: The physico-chemical properties of the basic protein encephalitogen from bovine spinal cord. *Arch. Biochem. Biophys.*, **129**, 468.

FIELD, E. J. & CASPARY, E. A. (1970) Lymphocyte sensitization. An *in vitro* test for cancer? *Lancet*, ii, 1137.

FLAVELL, D. J., GOEPEL, J., POTTER, C. W. & CARR, I. (1978) Cellular immunity to encephalitogenic factor as measured by macrophage migration inhibition during tumour induction and growth. *Br. J. Cancer*, **37**, 818.

FLAVELL, D. J. & POTTER, C. W. (1978) Cellular immunity to encephalitogenic factor in man as measured by the macrophage migration inhibition test: The effects of serum. *Br. J. Cancer*, **37**, 15.

FORRESTER, J. A., DANDO, P. M., SMITH, J. W. & TURBERVILLE, C. (1977) Failure to confirm the macrophage electrophoretic mobility test in cancer. *Br. J. Cancer*, **36**, 537.

GOLDSTONE, A. H., KERR, I. & IRVINE, W. J. (1973) The macrophage electrophoretic mobility test in cancer. *Clin. Exp. Immunol.*, **14**, 469.

HENSON, R. A., HOFFMAN, L. H. & URICH, H. (1965) Encephalomyelitis with carcinoma. *Brain*, **88**, 449.

LENNON, V. A., WILKS, A. V. & CARNEGIE, P. R. (1970) Immunologic properties of the main encephalitogenic peptide from the basic protein of myelin. *J. Immunol.*, **105**, 1123.

LIGHT, P. A., PREECE, P. W. & WALDRON, H. A. (1975) Studies with the macrophage migration inhibition test in patients with malignant disease. *Clin. Exp. Immunol.*, **22**, 279.

MCDERMOTT, J. R., CASPARY, E. A. & DICKINSON, J. P. (1974) Antigen cross reactivity in the macrophage electrophoretic mobility test. A study using cellular affinity chromatography. *Clin. Exp. Immunol.*, **17**, 103.

PATERSON, P. Y. (1969) Immune processes and infectious factors in central nervous system disease. *Ann. Rev. Med.*, **20**, 75.

PRITCHARD, J. A. V., MOORE, J. L., SUTHERLAND, W. H. & JOSLIN, C. A. F. (1972) Macrophage electrophoretic mobility (MEM) test for malignant disease. An independent conformation. *Lancet*, ii, 627.

REES, R. C. & POTTER, C. W. (1973) Immune response to adenovirus 12-induced antigens as measured *in vitro* by the macrophage migration inhibition (MMI) test. *Eur. J. Cancer*, **9**, 497.

SCHLAEPFER, W. W. (1974) Axonal degeneration in the sural nerves of cancer patients. *Cancer*, **34**, 371.

SHAW, C. M., ALVORD, E. C., KAKU, J. & KIES, M. W. (1965) Correlation of experimental allergic encephalomyelitis with delayed type skin sensitivity to specific homologous encephalitogen. *Ann. N.Y. Acad. Sci.*, **122,** 318.

SHELTON, J. B., POTTER, C. W. & CARR, I. (1975) Cellular immunity to myelin basic protein in man and in animal model systems as measured by the macrophage migration inhibition test. *Br. J. Cancer*, **31,** 528.

SWANBORG, R. H. (1970) The effect of the selective modification of tryptophan, lysine and arginine residues of basic protein on encephalitogenic activity. *J. Immunol.*, **105,** 863.

TRAUGOTT, U., STONE, S. H. & RAINE, C. S. (1978) Experimental allergic encephalomyelitis—migration of early T-cells from the circulation into the central nervous system. *J. Neurol. Sci.*, **36,** 55.